# Supplementary material for: Does the Intensity of Therapy Correspond to the Severity of Acute Respiratory Distress Syndrome (ARDS)?
Source: J Clin Med. 2024 Nov 23;13(23):7084. doi: 10.3390/jcm13237084 (PMC11642668; doi:10.3390/jcm13237084)
Supplement: Supplementary file 1 [file jcm-13-07084-s001.zip › jcm-3306660-supplementary.pdf]

Journal of
Clinical Medicine

MDPI

SUPPLEMENTARY MATERIALS

Supplementary Methods

Study Population.

The study population includes 291 patients with ARDS, enrolled between May 2003 and July 2024, from San Paolo University Hospital of Milan (Italy), University Medical Center Göttingen (Germany), Ospedale Maggiore Policlinico of Milan (Italy), Azienda Ospedaliera San Gerardo Monza (Italy) and Pontificia Universidad Catolica de Chile (Chile). Patients were classified based on their severity.

Mechanics Measurement:

The driving pressure (DP) was defined as:

$$Driving\ Pressure\ =\ Plateau\ Pressure - PEEP \quad \text{(S1)}$$

The mechanical power (MP) was computed as

$$MP = 0.098 \times RR \times \{V_T{}^2 \times \left[\frac{1}{2} \times E_{rs} + RR \times \frac{1 + I{:}E}{60 \times I{:}E} \times R_{aw}\right] + V_T \times PEEP\} \quad \text{(S2)}$$

where RR is the respiratory rate, V_T the tidal volume, E_{rs} the respiratory system elastance, I:E the ratio of inspiratory time to the expiratory time, R_{aw} the airways resistance and PEEP the positive end-expiratory pressure

Statistical Analysis.

Continuous variables are presented as means with standard deviations, while categorical variables are shown as percentages. Linear regression was used to assess the relationship between continuous variables. To compare groups, we used one-way ANOVA or the Kruskal-Wallis test for continuous data, depending on suitability, with Bonferroni correction. Mortality rates were analyzed by the Pearson's chi-square test.Post-hoc comparisons between pairs of groups were done using either Student's t-tests or Wilcoxon tests, as appropriate. A p-value of less than 0.05 was considered statistically significant .

SUPPLEMENTARY RESULTS

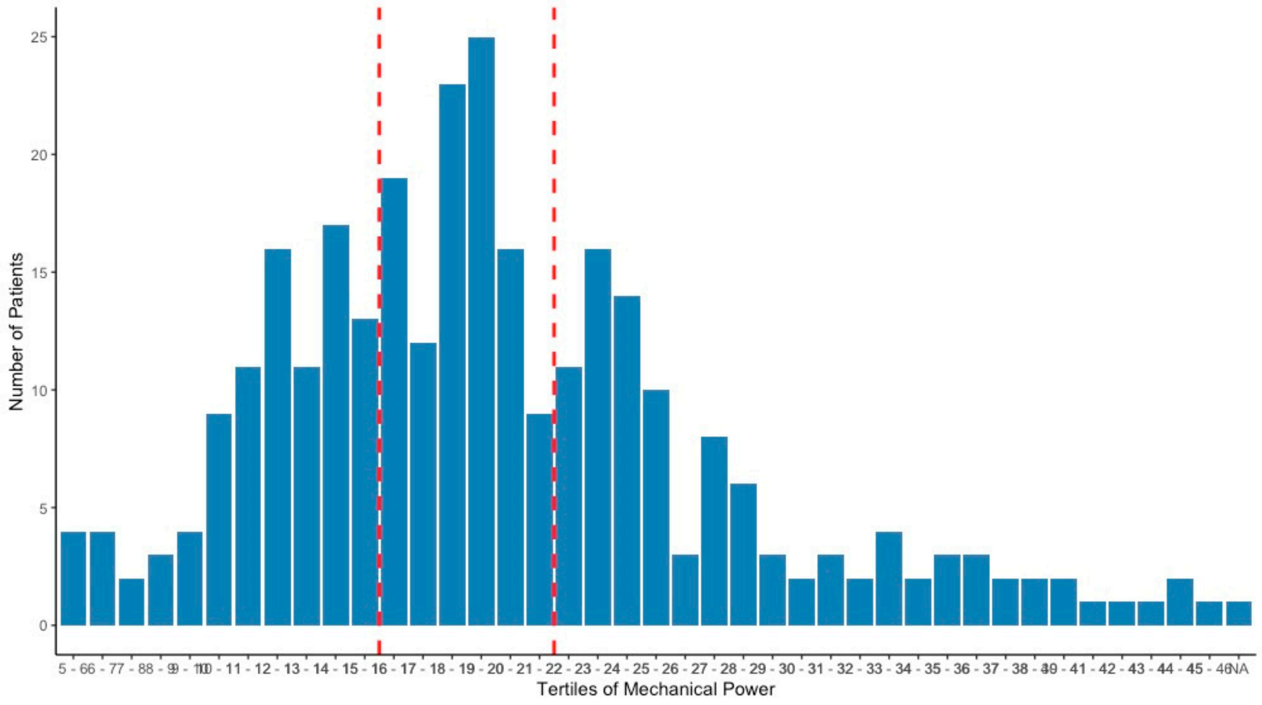

Figure S1. distribution of population across the three ARDS severity$_{MP}$ classes.

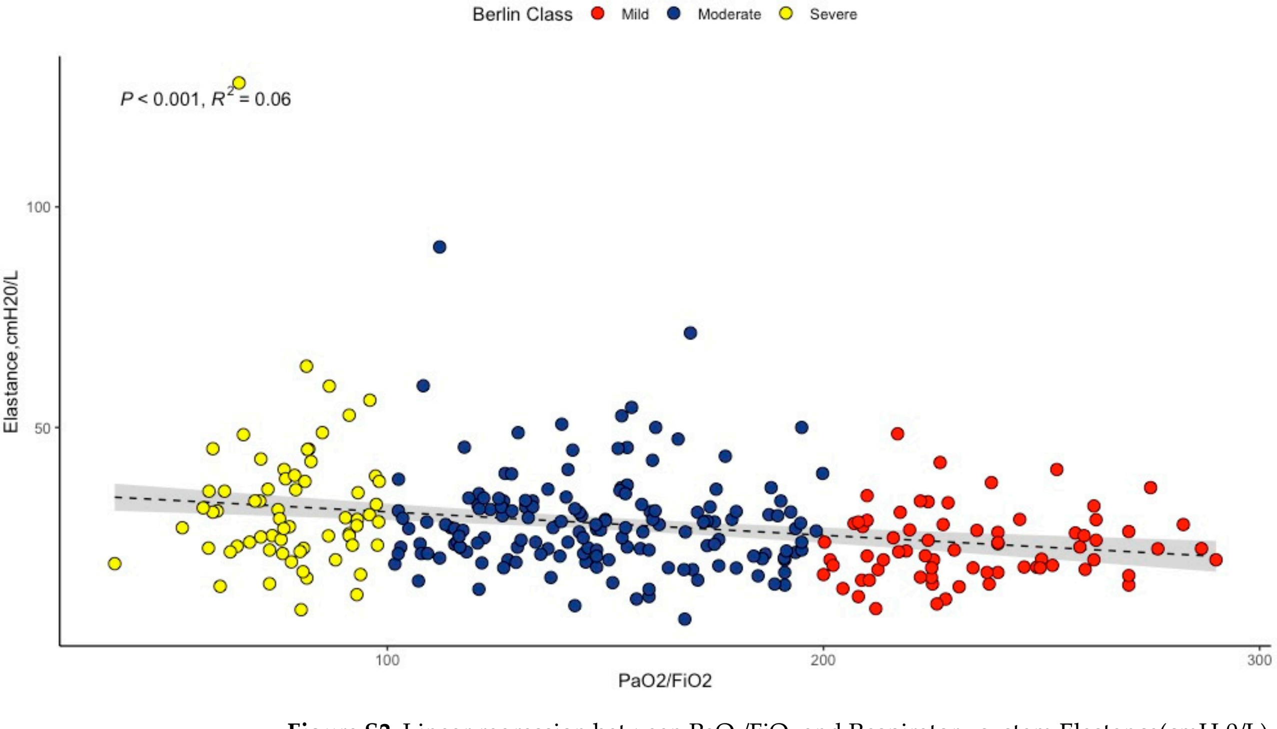

Figure S2. Linear regression between PaO_2/FiO_2 and Respiratory system Elastance(cmH20/L).

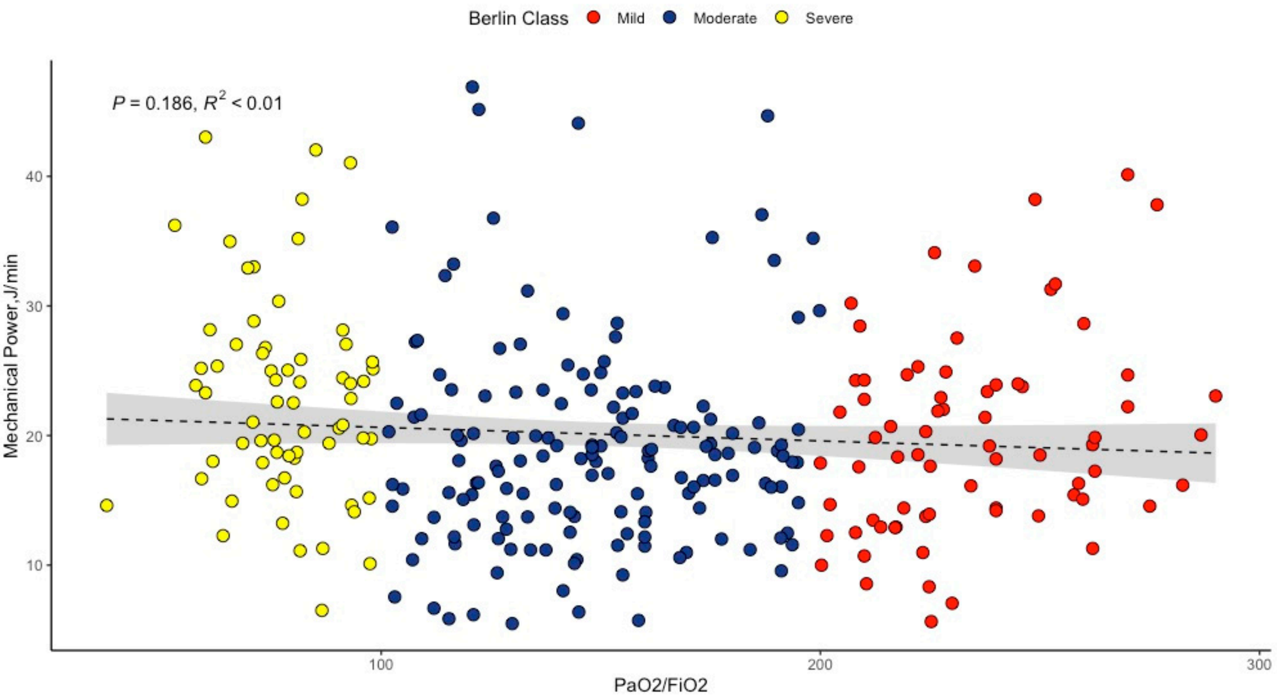

Figure S3. Linear regression between PaO_2/FiO_2 and Mechanical power(J/min).

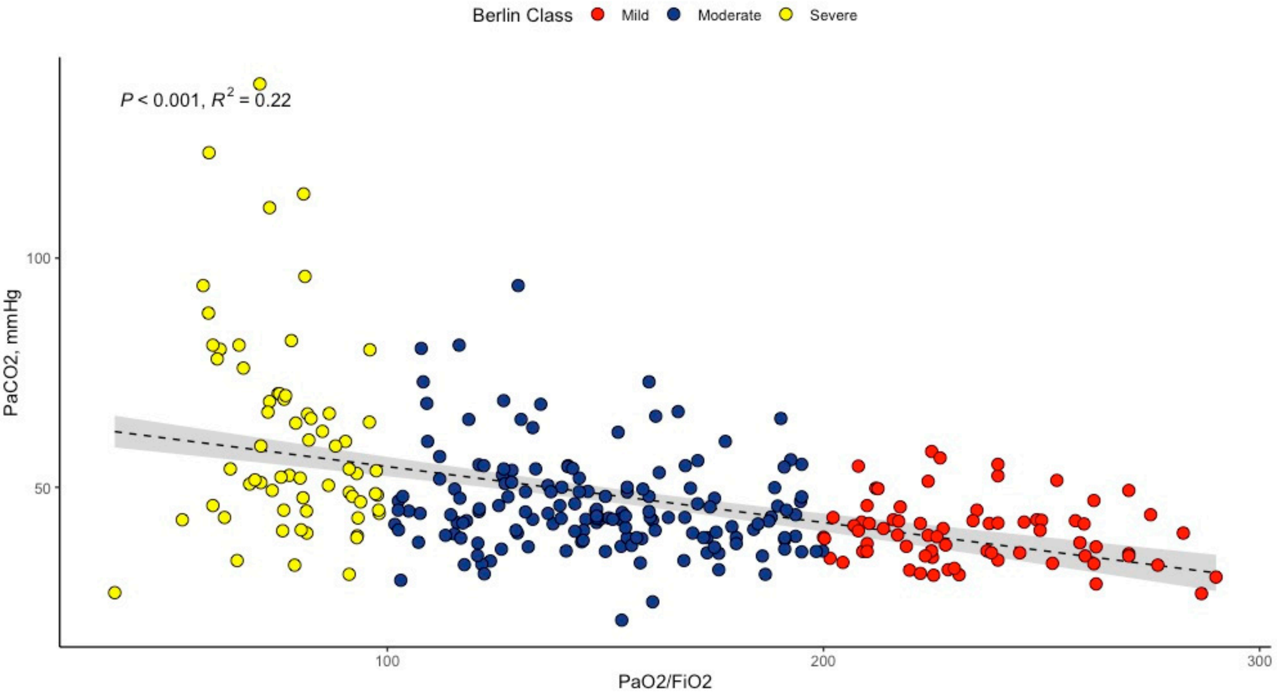

Figure S4. : Linear regression between PaO_2/FiO_2 and $PaCO_2$.

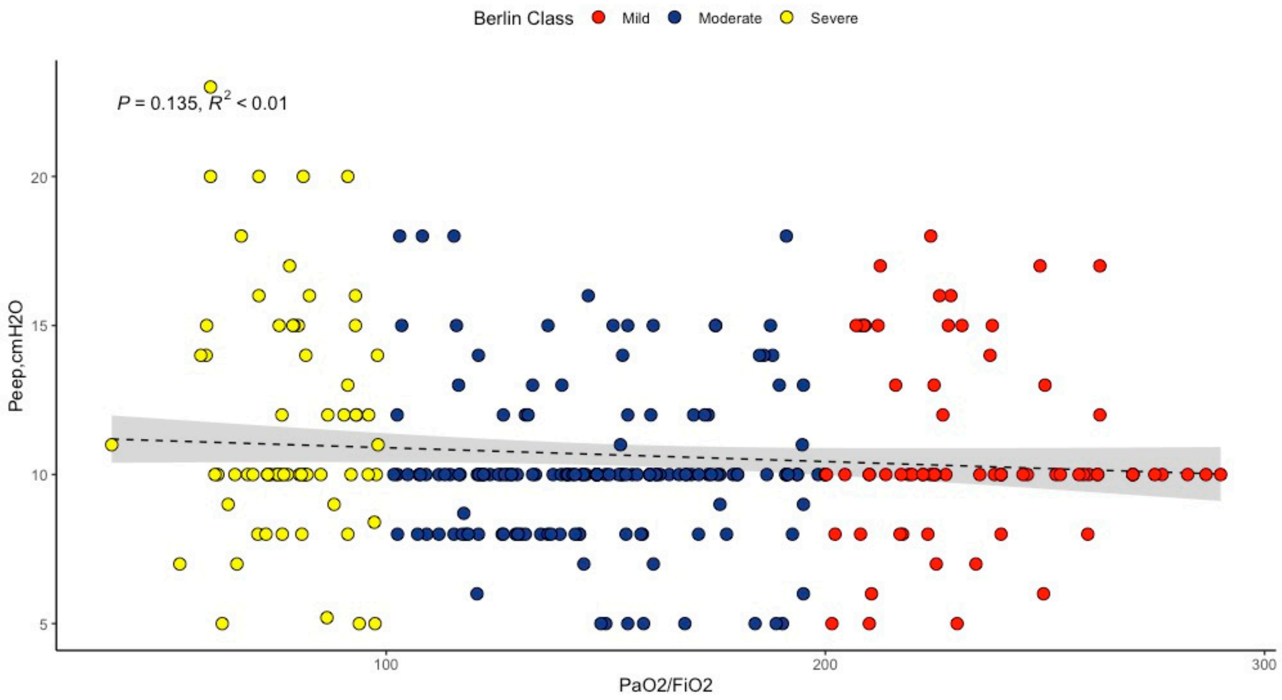

Figure S5. Linear regression between PaO_2/FiO_2 and Positive end-expiratory pressure(cmH_20).

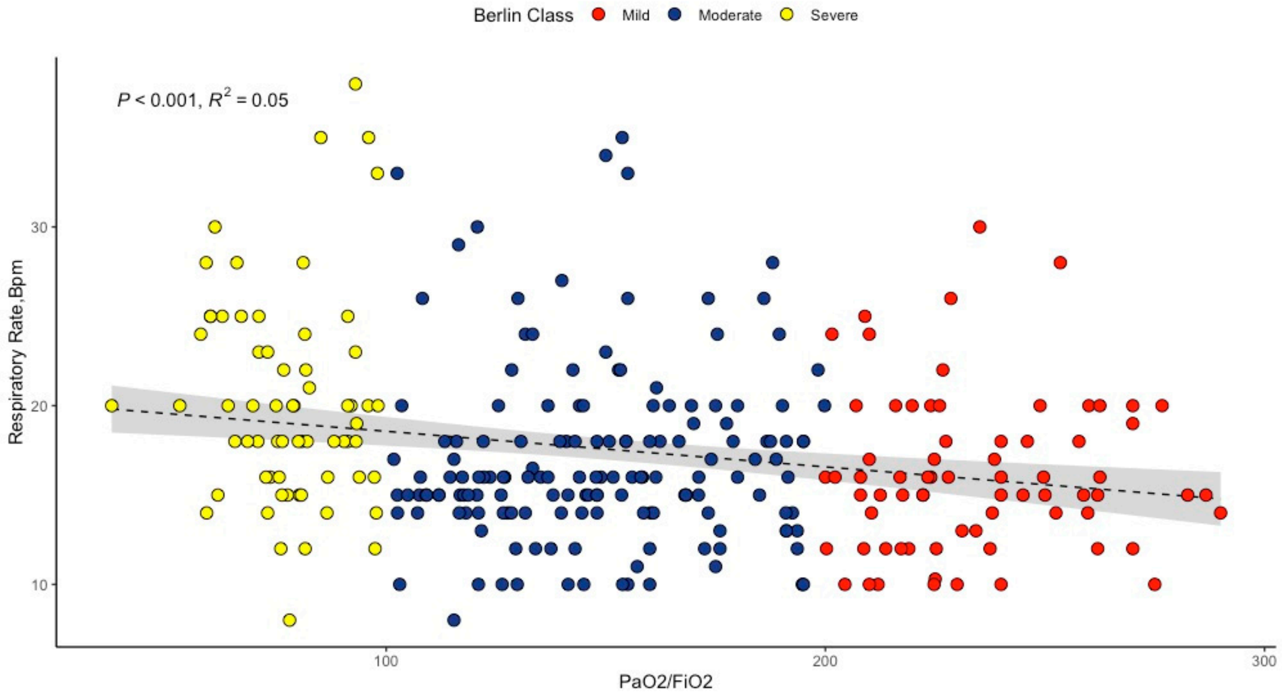

Figure S6. Linear regression between PaO_2/FiO_2 and respiratory rate(bpm).

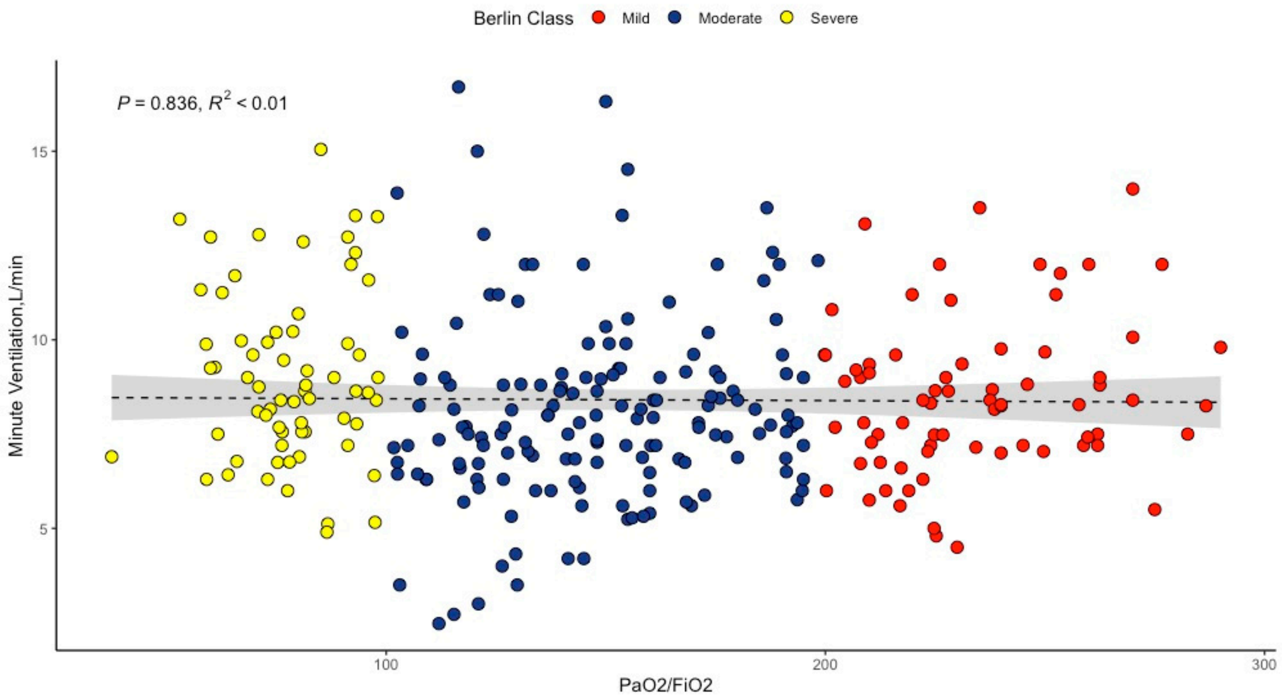

Figure S7. Linear regression between PaO_2/FiO_2 and Minute ventilation(L/min).

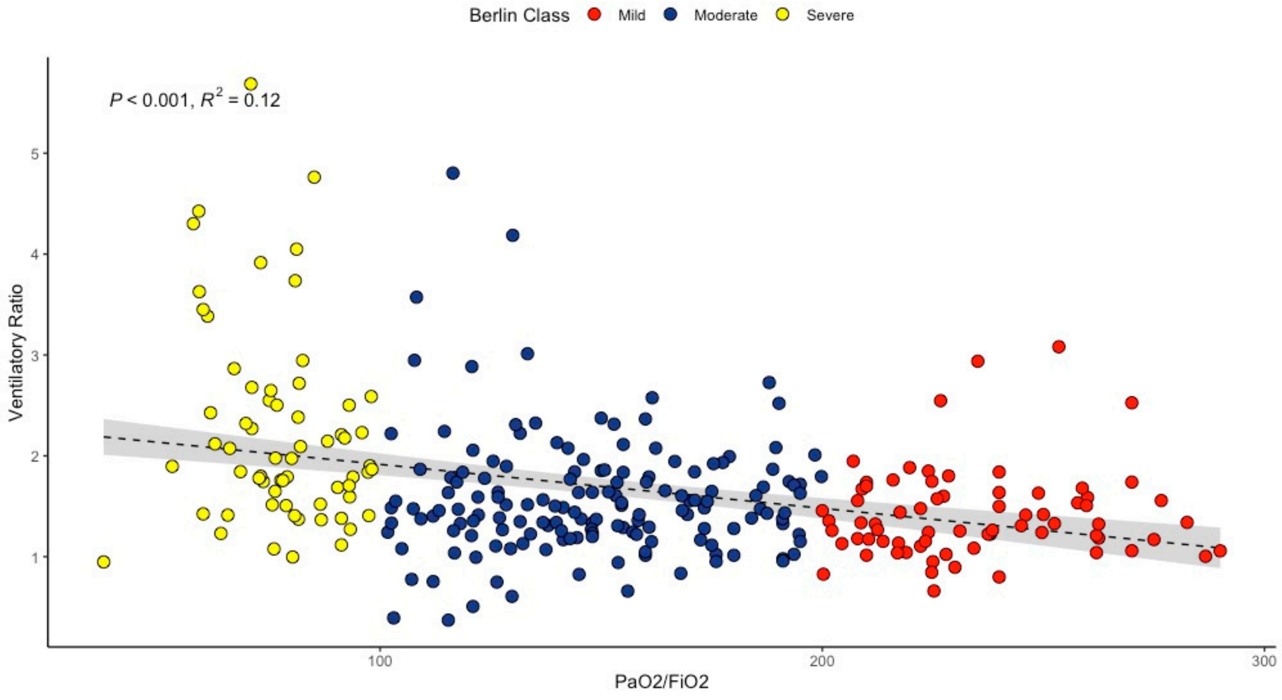

Figure S8. Linear regression between PaO_2/FiO_2 and Ventilatory ratio.

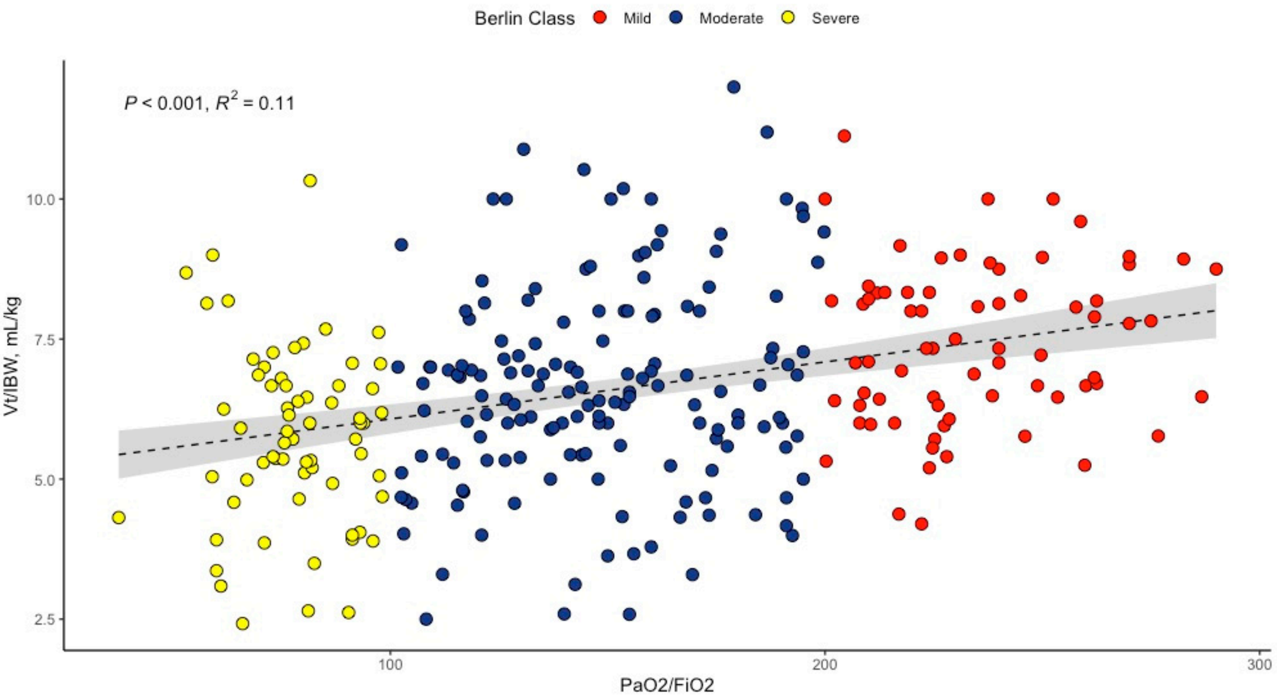

Figure S9. Linear regression between PaO_2/FiO_2 and tidal volume /IBW.

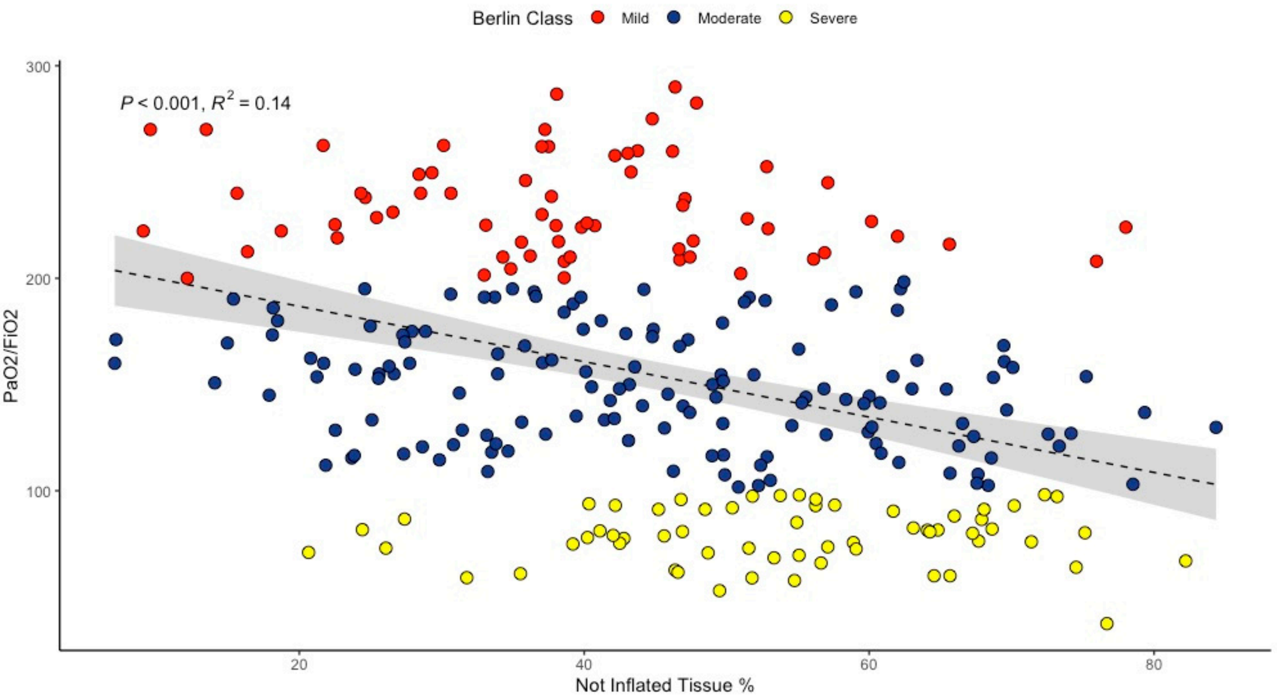

Figure S10. Linear regression between Not inflated tissue(Ct scan) and PaO_2/FiO_2 at 5 cmH_20 of peep.

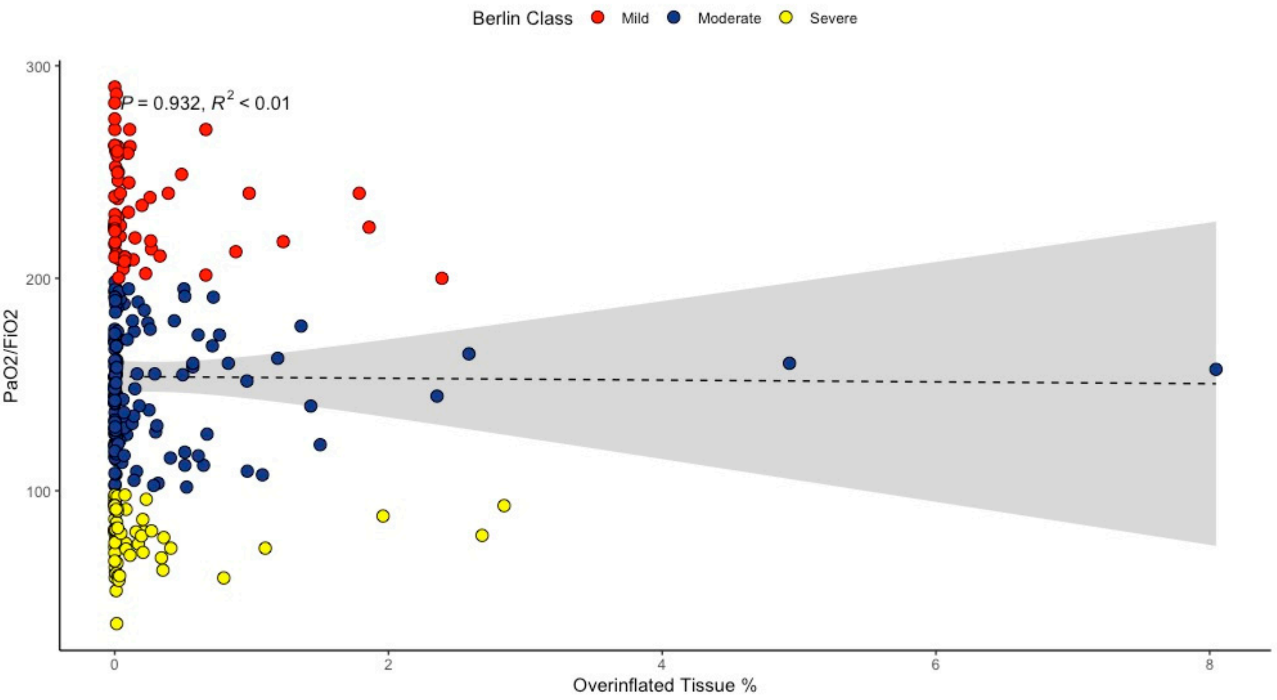

Figure S11. Linear regression between Overinflated tissue %(Ct scan) and PaO_2/FiO_2 at 5 cmH_20.

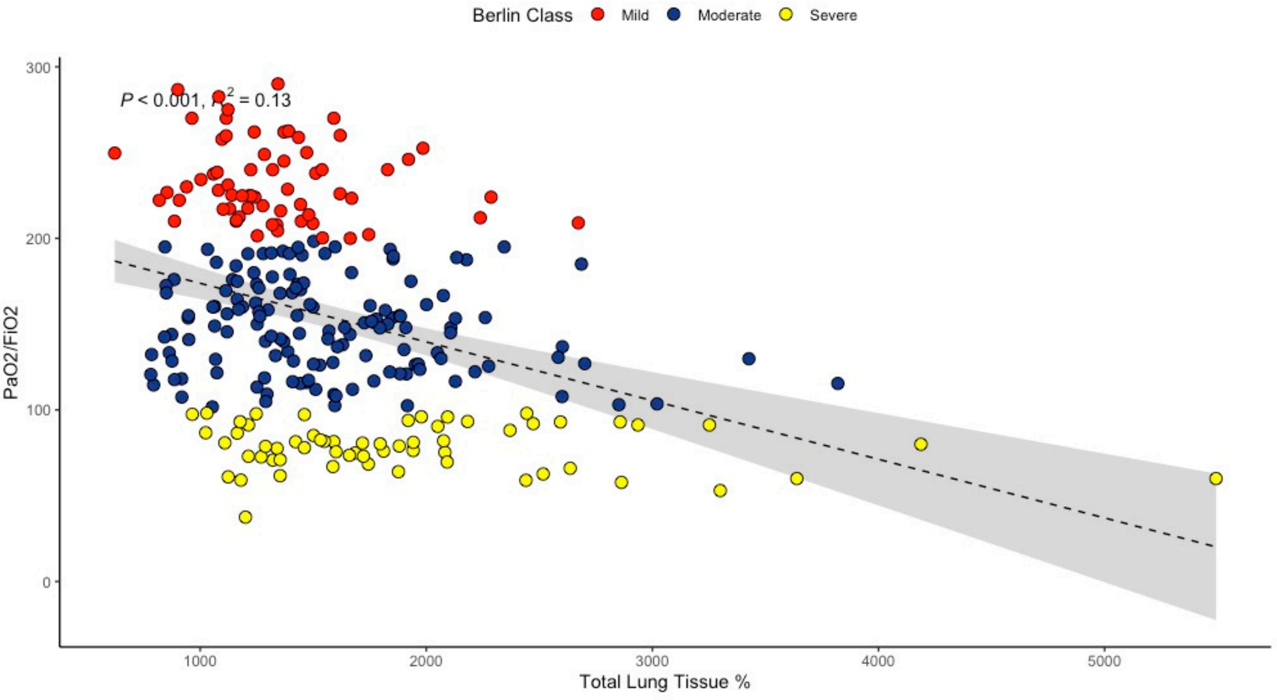

Figure S12. : Linear regression between Total lung tissue %(Ct scan) and PaO_2/FiO_2 at 5 cmH_20.

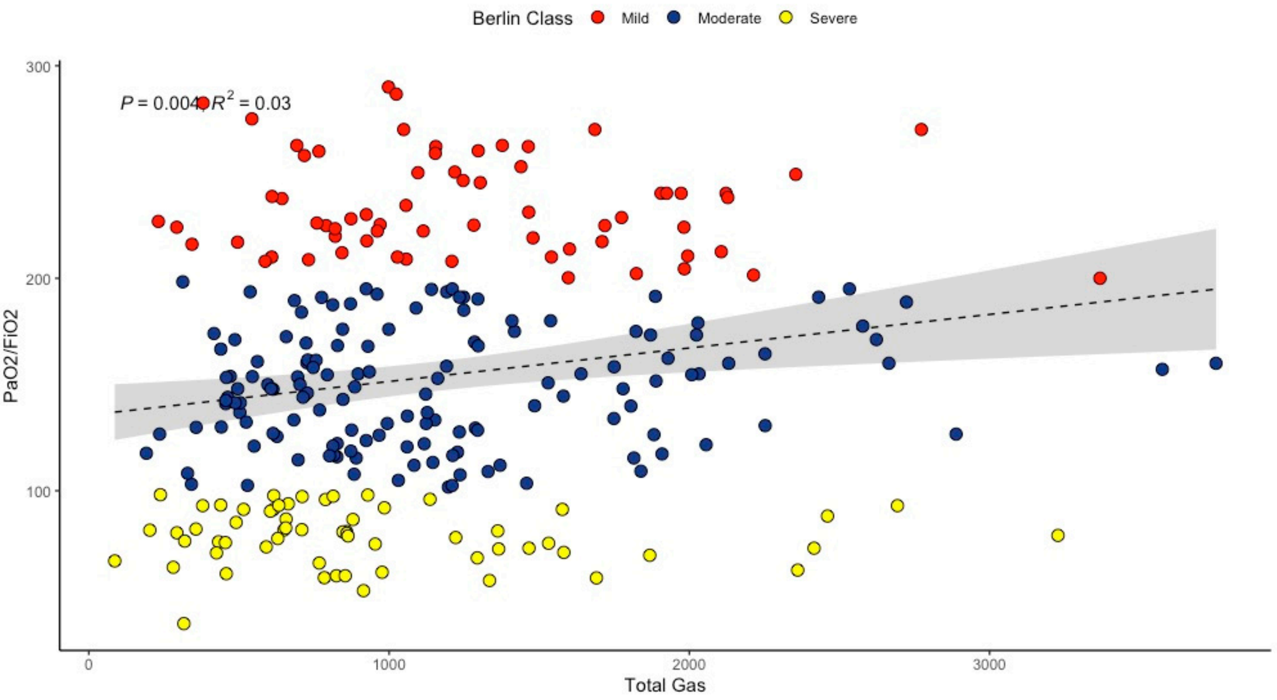

Figure S13. Linear regression between Total Gas (Ct scan) and PaO_2/FiO_2 at 5 cmH_20.

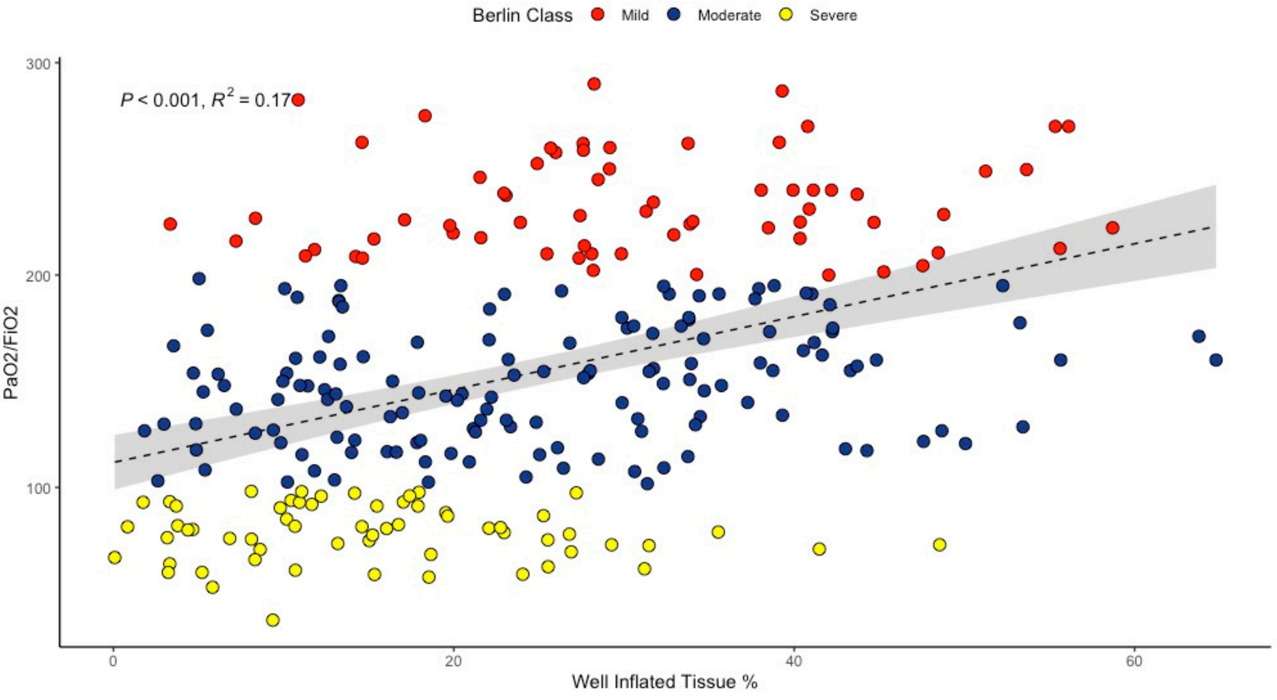

Figure S14. Linear regression between well-inflated tissue and total lung tissue at 5 cmH_2O of PEEP.

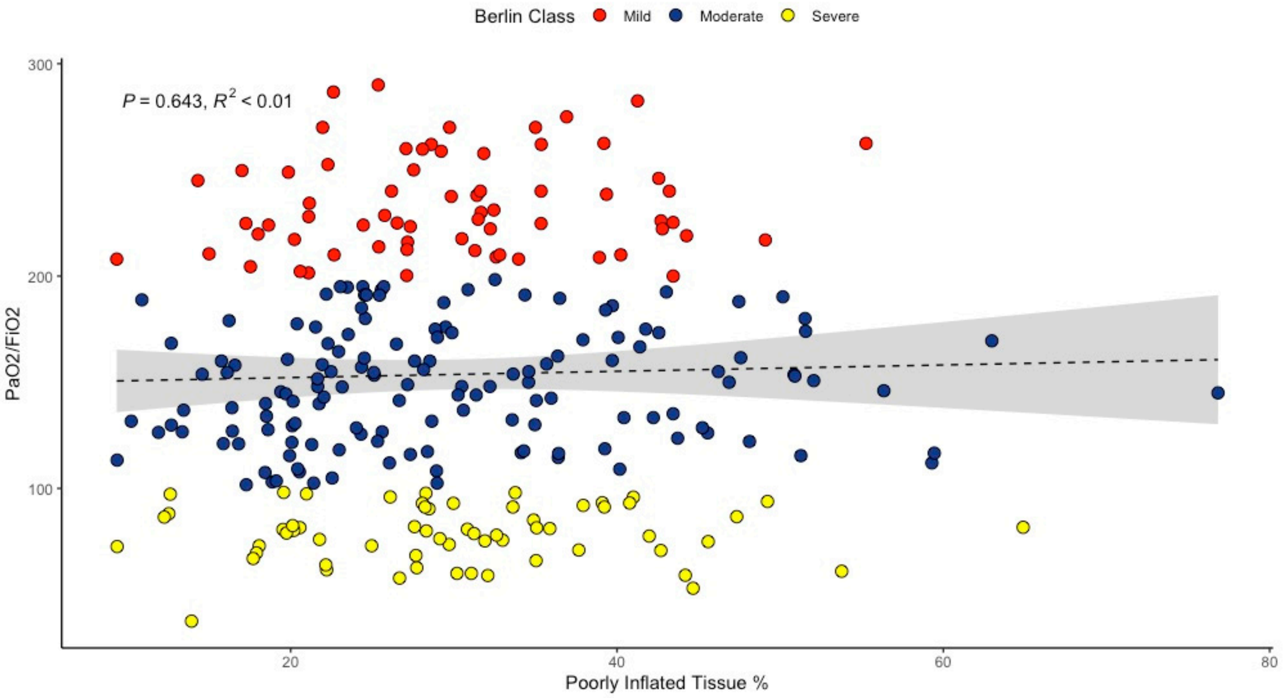

Figure S15. Linear regression between Poorly Inflated Tissue %(Ct scan) and PaO_2/FiO_2 at 5 cmH_20.

Equation of the logistic regression on mortality:

$$log\frac{P\ (Y=0)}{P\ (Y=1)} = \beta_0 + \beta_1 \cdot PF + \beta_2 \cdot MP(\frac{J}{\text{min}})$$
$$= 0.149918 - 0.007817 \cdot \text{PF} + 0.037830 \cdot \text{MP}\ (\frac{J}{\text{min}}) \quad \text{(S3)}$$

- P(Y=0) is the probability that the outcome **does not happen**,
- P(Y=1) is the probability of the outcome being "dead",
- β_0 is the intercept,
- β_1, is the coefficient for the variable "PF",
- β_2 is the coefficient for the variable "Mechanical Power(J/min)".

Overall, this logistic regression model reveals how changes in the PaO_2/FiO_2 ratio and Mechanical Power influence the odds of survival versus mortality. Specifically, better oxygenation (higher PF) is linked to improved survival chances, while higher Mechanical Power is linked to an increased risk of death.

Other variables independently associated with mortality:

Logistic Regression analysis	
$log\frac{P\ (Y=0)}{P\ (Y=1)} = -0.65 - 0.007 \cdot \text{P/F} + 0.11 \cdot \text{Driving Pressure}$	p-value P/F: <0.001<br>p-value ΔP: <0.001
$log\frac{P\ (Y=0)}{P\ (Y=1)} = -0.17 - 0.006 \cdot \text{P/F} + 0.49 \cdot \text{Ventilatory Ratio}$	p-value P/F:0.002<br>p-value Ventilatory Ratio:0.01
$log\frac{P\ (Y=0)}{P\ (Y=1)} = -0.64 - 0.0079 \cdot \frac{\text{P}}{\text{F}} + 0.039 \cdot \text{Ventiltory Ratio}$	p-value P/F: <0.001<br>p-value Ventilatory ratio:<0.001
